# Determinants of diagnostic yield in a multi-ethnic Asian inherited retinal disease cohort

**DOI:** 10.1038/s41431-025-01833-w

**Published:** 2025-03-21

**Authors:** Jane Andrea Lieviant, Choi Mun Chan, Yasmin Bylstra, Kanika Jain, Jing Xian Teo, Wan Wan Lim, Sylvia Kam, Tang Wei Chao, Nellie Chai Bin Siew, Sonia Davila, Eranga Nishanthie Vithana, Ranjana Sanjay Mathur, Tien-En Tan, Patrick Tan, Saumya S. Jamuar, Beau James Fenner, Weng Khong Lim

**Affiliations:** 1 SingHealth Duke-NUS Institute of Precision Medicine, 169609 Singapore, Singapore; 2 Singapore National Eye Center, 168751 Singapore, Singapore; 3 SingHealth Duke-NUS Genomic Medicine Centre, 168582 Singapore, Singapore; 4 Genome Institute of Singapore, Agency for Science, Technology and Research, 138672 Singapore, Singapore; 5 KK Women’s and Children’s Hospital, Singapore, Singapore; 6 Singapore Eye Research Institute, Singapore, Singapore; 7 Duke-NUS Medical School, 169857 Singapore, Singapore; 8 Cancer & Stem Cell Biology Program, Duke-NUS Medical School, 169857 Singapore, Singapore; 9 Cancer Science Institute of Singapore, National University of Singapore, 117599 Singapore, Singapore; 10 Department of Pediatrics, Genetics Service, KK Women’s and Children’s Hospital, 229899 Singapore, Singapore; 11 Paediatric Academic Clinical Program, Duke-NUS Medical School, 169857 Singapore, Singapore

**Keywords:** Disease genetics, Computational biology and bioinformatics

## Abstract

As the discovery of new genes causing inherited retinal disease (IRD) has plateaued, we look to other factors which could be used to maximize diagnostic yield. We analyzed whole-exome sequencing (WES) data from 506 IRD probands, focusing on the interplay between diagnostic yield, age of symptom onset or diagnosis, family history, and initial clinical diagnosis. The cohort’s overall diagnostic yield was 49.2%. Diagnostic yield was negatively correlated with the age of symptom onset and positively correlated with the number of affected family members. Diseases with distinctive clinical presentations such as Bietti crystalline dystrophy (BCD) or Leber congenital amaurosis (LCA) were more reliably diagnosed than more common and heterogeneous diseases like retinitis pigmentosa (RP) and cone-rod dystrophy (CRD). Recurrent genes and variants in this Chinese-majority cohort resemble those found in Chinese cohort studies but differ from populations of European descent, with implications for the design and prioritization of gene therapies. These insights may help optimize the diagnostic utility of genetic testing for IRDs, enhance the delivery of genetic counseling for patients, and guide the development of more inclusive targeted therapies.

## Introduction

Inherited retinal diseases (IRDs) are a heterogeneous group of eye diseases caused by pathogenic variants in genes associated with the structure and function of retinal cells. Estimated to have a worldwide prevalence of 0.07% [1], IRDs are thought to affect 0.1% of Singaporeans [2, 3] and may manifest in non-syndromic (e.g. Bietti crystalline dystrophy [BCD], Stargardt disease [STGD]) or syndromic (e.g. Usher syndrome [USH], Bardet-Biedl syndrome [BBS]) forms. Furthermore, depending on their causal genes and specific variants, IRDs can be inherited in autosomal dominant, autosomal recessive, X-linked, or mitochondrial patterns.

The genetic heterogeneity of IRDs poses a challenge when it comes to making an accurate molecular genetic diagnosis, compounded by the fact that many IRDs, particularly in their later stages, have overlapping phenotypes. Furthermore, despite advances brought forth by next-generation sequencing (NGS), the diagnostic yield for IRDs remains suboptimal, especially in underrepresented populations. For example, the mean diagnostic yield observed in East Asian cohorts is only 52.2% compared to a global mean diagnostic yield of 61.3% [4–13]. One possible cause for this difference is the relative lack of representation of these populations in ophthalmic genetic research and population genetic databases, which has consequent implications for the accuracy of classification of variant pathogenicity. Additionally, we hypothesize that factors such as age at diagnosis, family history and specific clinical phenotypes may influence diagnostic yield.

The importance of obtaining an accurate molecular diagnosis has recently been reinforced by the emergence of gene therapies and other targeted therapies that are being developed for specific causative variants or genes, and having a confirmed molecular diagnosis is a prerequisite for such treatments. This is on top of the other established benefits of having a molecular diagnosis such as more precise disease management, cascade testing for family members, and reproductive planning. Although the cost of sequencing has fallen in recent years, the total cost of delivering genetic medicine, which includes genetic counseling, clinical geneticists, variant scientists among others, remains high and poses a significant challenge especially in resource-limited settings such as low- or middle-income countries and public healthcare systems. Here, we analyze WES data from a multi-ethnic Asian cohort of 506 probands with suspected IRD to identify determinants of diagnostic yield, with a goal of improving prioritization of patients for genetic testing, providing more accurate pretest counseling and management of patient expectations regarding the likelihood of receiving a molecular diagnosis, and increasing access to emerging targeted therapies.

## Methods

### Patient recruitment and DNA sequencing

Participants were recruited prospectively from the Singapore National Eye Centre, Singapore, during the period from January 2018 to September 2023. Patients diagnosed with IRD were enrolled during their clinic visit. Diagnoses were made by retinal specialists based on clinical history, physical examination, ophthalmic imaging, and psychophysical testing. This study was approved by the SingHealth Institutional Review Board (SHF-SNEC 0920-4) and conducted in accordance with the ethical standards of the 1964 Declaration of Helsinki and its later amendments. Informed consent was obtained from all participants.

DNA extracted from participant whole-blood samples were enriched for exonic regions using the KAPA HyperExome kit and then sequenced on the Illumina NovaSeq 6000 platform. A subset of participants with inconclusive findings but whose clinical presentations were suggestive of RPGR-associated retinal degeneration or a potential second intronic pathogenic variant, were sent to Molecular Vision Lab for sequencing on the MVL Vision Panel, which covers *RPGR* ORF15 and known deep intronic variants. Where necessary, Sanger sequencing to validate candidate variants was performed with the BigDye Terminator Cycle Sequencing kit (Applied Biosystems, Foster City, CA) and the 3130 Genetic Analyzer (Applied Biosystems).

### Variant calling

Sequencing reads were analyzed using a standardized bioinformatics pipeline that involved alignment to the human reference genome (hg38) using Burrows-Wheeler Aligner (v0.7.17) [14] followed the by Genome Analysis Toolkit (v4.0.6.0) [15] best practices workflow to produce a jointly genotyped variant call file. Annotation for single nucleotide variants was performed using Ensembl Variant Effect Predictor (release 100.0) [16] to include information such as overlapping genes, consequence type, Human Genome Variation Society (HGVS) [17] nomenclature for DNA and protein alterations, population allele frequencies and in silico pathogenicity prediction scores from REVEL [18], PrimateAI [19] and SpliceAI [20]. Copy number variants (CNVs) were identified using ExomeDepth [21] under default settings. The results were filtered to identify deletions in IRD genes. Deletions with a read count ratio (RR) of between 0.3 and 0.7 were considered heterozygous and those with a RR of < 0.1 were considered to homozygous/hemizygous. Duplications were retained only if RR > 1.3. All candidate CNVs had their ExomeDepth plots visually inspected to avoid false positives. Genetic variant calls were made by comparison with SG10K, a Singapore population-based genome reference database [22], with additional reference to the gnomAD genomic database [23] and ClinVar for variants absent from SG10K. Variant phase was established by direct sequencing of the relevant variants in one or more unaffected first-degree relatives.

### Variant classification

We started by identifying candidate variants seen in a panel of 410 IRD-associated genes derived from PanelApp [24] (Supplementary Table 1). The pathogenicity of candidate variants was evaluated and assigned numerical scores according to ACMG/AMP (American College of Medical Genetics/Association for Molecular Pathology) guidelines [17]. Each criterion was assessed and assigned a score depending on the strength of the evidence; 8 for Very Strong, 4 for Strong, 2 for Moderate and 1 for Supporting. Specific considerations for each criterion are available in Supplementary Notes 1. The evidence scores were then tabulated and a final classification assigned ($x \, < \, 0$ as Benign/Likely Benign; $0\le x \, < \, 5$ as VUS, $x=5$ as VUS-FP, $6\le x \, < \, 10$ as Likely Pathogenic, and $x\ge 10$ as Pathogenic) where VUS stands for variant of uncertain significance and VUS-FP as VUS-favouring pathogenic. Cases were considered fully solved if identified variant(s) were classified as P/LP. Otherwise, cases with variant(s) classified as VUS-FP or VUS may be considered probably solved if certain criteria were met. For further details please see Supplementary Note 1. For patients with pairs of variants in recessive genes that had parental DNA available, we sequenced the variants in both parents to confirm that they are in trans. Our analysis showed that 90% of cases were in trans, providing a high degree of confidence in the validity of the probably solved cases. Phasing and segregation data is also available in Supplementary Table 1.

### Statistical analysis

All statistical analyses were performed with R [25] (v4.4). Participant data, gene- and variant-level frequencies were tabulated with descriptive statistics. We used two-sided Fisher’s exact test to compare the proportions of categorical groups, and Wilcoxon’s rank-sum test for comparing continuous variables. Binomial logistic regression was used for the comparison of age of diagnosis against molecular diagnosis. We ran kinship analysis using KING (v2.3.2) [26] and PLINK [27] on the WES data to analyze the capture rate of the family history and kinship data.

## Results

### Cohort characteristics

Out of 589 participants that were sequenced, we identified and excluded related individuals through a combination of self-reported familial relationships and genetic kinship analysis, yielding a final cohort of 506 unrelated individuals (Table 1). The cohort comprised of predominantly Chinese ancestry (406/506, 80.2%), followed by those of Indian (47/506, 9.3%) and Malay (40/506, 7.9%) ancestry, and the remaining 2.6% (13/506) of other ancestries. 235 patients (46.4%) were female, and 271 patients (53.6%) were male. At time of examination, patient age ranged from 6–91, with a median of 54, whereas age of symptom onset or diagnosis (where available) ranged from 0–79 with a median of 31. Approximately a third (166/506, 32.8%) of patients reported a family history of IRD.

**Table 1:** Table of cohort demographics and status of molecular diagnosis.

	Female	Male	Total
**Ancestry**			
**Chinese**	188 (37.15%)	218 (43.08%)	406 (80.24%)
**Indian**	24 (4.74%)	23 (4.55%)	47 (9.29%)
**Malay**	20 (3.95%)	20 (3.95%)	40 (7.91%)
**Other**	3 (0.59%)	10 (1.98%)	13 (2.57%)
**Molecular Diagnosis**			
**Solved**	78 (15.42%)	98 (19.37%)	176 (34.78%)
**Probably solved**	32 (6.32%)	38 (7.51%)	70 (13.83%)
**Not solved**	125 (24.70%)	135 (26.68%)	260 (51.38%)
**Family History**			
**Present**	78 (15.42%)	88 (17.39%)	166 (32.81%)
**None**	157 (31.03%)	183 (36.17%)	340 (67.19%)
**Age (mean** **±** **standard deviation)**			
**Age**	6–91 (59.0 ± 18.1)	9–89 (57.0 ± 19.1)	6–91 (54.1 ± 18.6)
**Age of Diagnosis**	0–79 (36.0 ± 20.3)	0–77 (25.0 ± 20.6)	0–79 (32.1 ± 20.7)

### Determinants of diagnostic yield

**Fig. 1:**
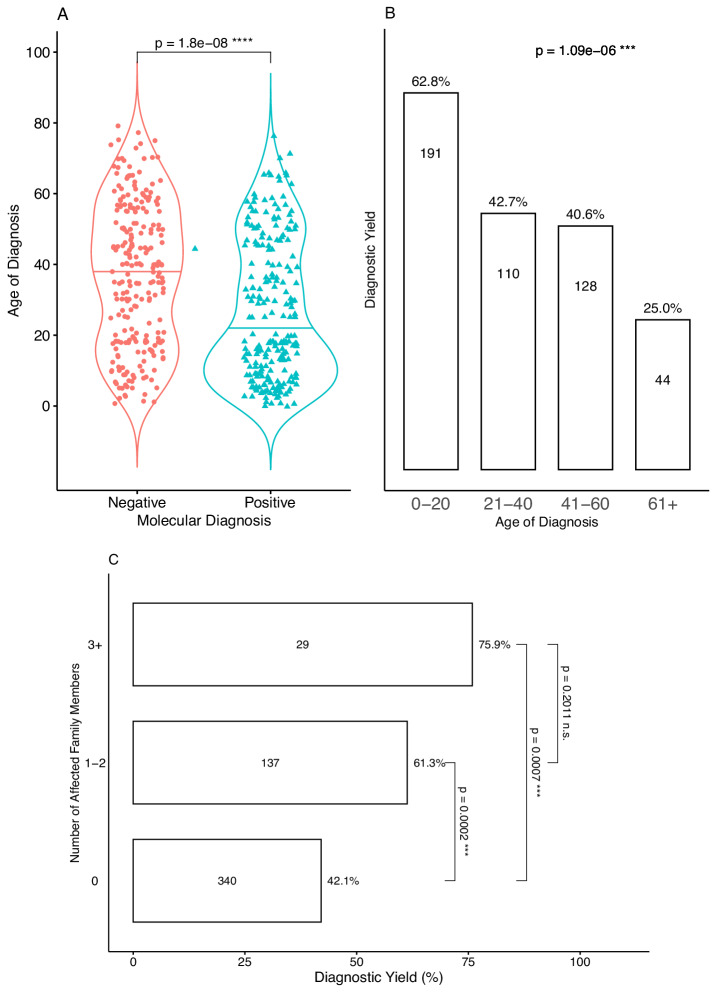
Diagnostic yield plotted against age of diagnosis and number of affected family members. **A** distribution of age of diagnosis/onset in patients who received molecular diagnosis (positive) and not (negative). Median age of diagnosis of the population is represented by horizontal line at 31. *P*-value is calculated using the Wilcoxon rank-sum test. **B** The diagnostic yield of the IRD cohort grouped by age of diagnosis. Numbers within each bar represent the total number of patients within the group. **C** Diagnostic yield of patients grouped by the self-reported number of family members with similar phenotypes. Numbers within boxes are the number of patients within each group.

Approximately half of the probands were considered solved (249/506, 49.21%), of which 35.18% (178/506) were considered fully solved and 14.03% (71/506) probably solved. As with most genetic disorders, IRDs tend to have a younger age of onset compared to sporadic late-onset forms of retinal degeneration [28]. When comparing the mean age of disease onset or diagnosis in people who received molecular diagnosis and those who did not, we observed 20 years of difference in median (19 vs. 39, *p* = 1.8e-08, Fig. 1A) and a significant negative correlation (β = −0.026) between molecular diagnosis and age of onset. The difference became starker when we compared patients with the youngest and oldest age of diagnosis, with those diagnosed < 20 years having a 62.8% diagnostic yield (OR 1.69), compared to a yield of only 25.0% (OR 0.33) among those diagnosed > 60 years (*p* = 1.09e-06, Fig. 1B), or a 5.07-fold increase in odds ratio.

Our previous study and others have shown that having a positive family history was associated with an increased likelihood of identifying pathogenic variants in relevant genes [4, 16–18]. To explore this effect in our IRD cohort, we compared diagnostic yield among patients that reported family history of IRD and those that did not (Fig. 1C). Compared to individuals with no reported family history of IRD (42.1% yield), individuals reporting one or two family members with IRD were 1.46-fold more likely to receive a genetic diagnosis (61.3% yield, CI = 1.43–3.35, *p* = 0.0002). This is slightly pronounced in individuals reporting three or more family members with IRD (75.9% yield, OR = 1.80, CI = 1.72–12.29, *p* = 0.0007).

We next asked if we could combine family history and age of diagnosis to identify a subset of high-risk patients that would benefit most from genetic testing, and found that patients with self-reported family history of IRD and age of diagnosis below 40 years had a diagnostic yield of 69.52%, or 3.07-fold increase in likelihood (CI = 1.89–5.04, *p* = 1.5e-06) than the group that did not fulfill both criteria; compared to the cohort-wide yield it is increased by 1.41-fold. However, the difference in diagnostic yield between ancestries — Chinese (50.5%), Indian (55.3%), Malay (42.5%), and other (61.5%) — was not significant (Fisher’s two-sided exact test *p* = 0.604).

**Fig. 2:**
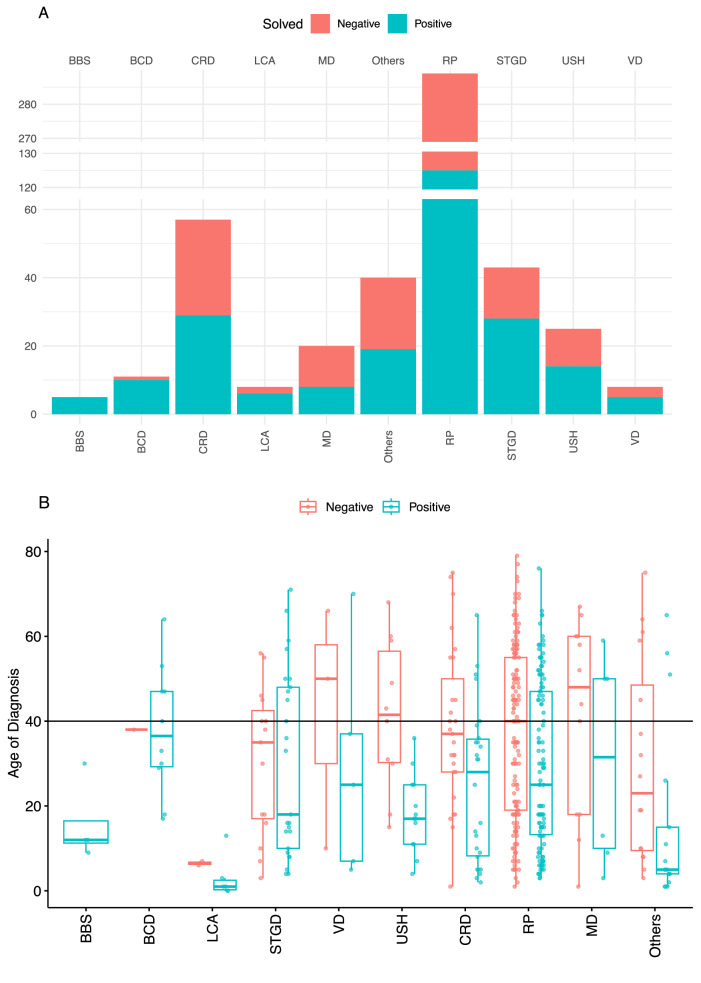
Diagnostic yield and age of diagnosis in patients grouped by their clinical phenotype. **A** Overall diagnosis yield based on phenotype. The same abbreviations are used throughout the other figures in this paper. BBS Bardet-Biedl syndrome; BCD - Bietti crystalline corneoretinal dystrophy; LCA Leber congenital amaurosis; RS retinoschisis; STGD Stargardt and Stargardt-like retinal dystrophy, including ABCA4-related phenotypes; VD Vitelliform dystrophy; USH Usher syndrome; MD macular dystrophy; CRD cone or cone-rod dystrophy; RP retinitis pigmentosa. Other diagnoses with less than 5 patients are grouped together as ‘Others’ for clarity. **B** Age of diagnosis distribution based on molecular diagnosis.

Diagnostic yield varied across the various clinical phenotypes as defined by initial clinical diagnosis (Table 2). Certain conditions such as BBS, BCD and Leber congenital amaurosis (LCA) had a very high yield, with all BBS cases being solved (Fig. 2A). High yield conditions tended to be rare recessive IRDs that have distinctive features, such as BBS which presents with multi organ system involvement, BCD associated with recognizable crystalline retinal deposits and LCA which has severe early onset. Conversely, more common diagnoses such as retinitis pigmentosa (RP) and cone-rod dystrophy (CRD) had lower diagnostic yields, likely due to their non-syndromic nature, greater genetic heterogeneity (Table 2), as well as the tendency for a recessive inheritance pattern. We observed a general trend of mean age at diagnosis being lower in patients who received a molecular diagnosis compared to those that did not (Fig. 2B).

**Table 2:** Table of genotypes associated with the initial clinical phenotype. Phenotypes are not adjusted to fit the genotypes.

Phenotype	Probands Diagnosed (%)	Gene (Probands)
Retinitis pigmentosa	124/289 (43%)	*USH2A (30), EYS (28), RHO (9), RPGR (7), PRPH2 (6), RP1 (5), MFSD8 (3), CRX (2), IFT140 (2), PDE6A (2), PDE6B (2), RP1L1 (2), ABCC6 (1), C21orf2 (1), CHM (1), CLN3 (1), CNGA1 (1), CYP4V2 (1), FAM161A (1), HK1 (1), IMPDH1 (1), KLHL7 (1), MT-ATP6 (1), MYO7A (1), NRL (1), PCDH15 (1), PRPF3 (1), PRPF31 (1), REEP6 (1), RLBP1 (1), RP2 (1), SLC24A1 (1), SNRNP200 (1), SPATA7 (1), TOPORS (1), TULP1 (1), WDR19 (1), ZNF408 (1)*
Cone/cone-rod dystrophy	29/57 (51%)	*GUCY2D (6), ABCA4 (3), CRX (3), PRPH2 (3), PROM1 (2), C21orf2 (1), CACNA2D4 (1), CNGA3 (1), CRB1 (1), EYS (1), HK1 (1), MYO7A (1), RCBTB1 (1), RP1 (1), RP2 (1), RPGR (1), TTLL5 (1)*
Macular dystrophy	8/20 (40%)	*ABCA4 (1), CFH (1), CNGB3 (1), CRX (1), PROM1 (1), PRPH2 (1), RDH12 (1), RP1 (1)*
Usher syndrome	14/25 (56%)	*USH2A (10), ADGRV1 (1), COL2A1 (1), EYS (1), PEX6 (1)*
Bardet-Biedl syndrome	5/5 (100%)	*BBS2 (2), ARL6 (1), BBS10 (1), MKKS (1)*
Leber congenital amaurosis	6/8 (75%)	*CRB1 (4), PROM1 (1), RPGRIP1 (1)*
Stargardt disease	27/43 (63%)	*ABCA4 (24), PRPH2 (2), RP1L1 (1)*
Bietti crystalline corneoretinal dystrophy	10/11 (91%)	*CYP4V2 (9), PRPH2 (1)*
Knobloch syndrome	1/1 (100%)	*COL18A1 (1)*
Achromatopsia	1/1 (100%)	*CNGA3 (1)*
Alstrom syndrome	1/1 (100%)	*ALMS1 (1)*
Occult macular dystrophy	1/2 (50%)	*RP1L1 (1)*
PPRCA	1/3 (33%)	*RDH12 (1)*
Pattern dystrophy	3/3 (100%)	*PRPH2 (3)*
Pseudoxanthoma elasticum	1/1 (100%)	*ABCC6 (1)*
Vitelliform dystrophy	5/8 (62%)	*BEST1 (5)*
enhanced S-cone syndrome	1/1 (100%)	*NR2E3 (1)*
Foveal hypoplasia	1/1 (100%)	*PAX6 (1)*
Choroideremia	3/3 (100%)	*CHM (3)*
Retinoschisis	4/7 (57%)	*RS1 (4)*

### Recurrent variants

**Fig. 3:**
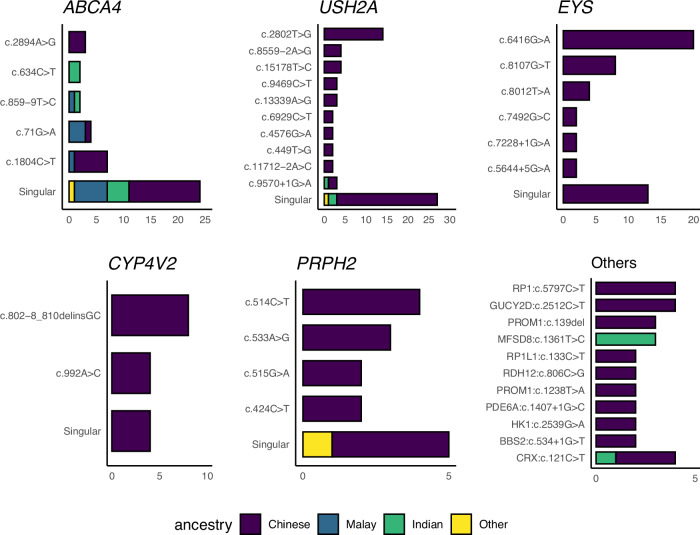
Variant frequency in the five most common pathogenic genes and others of note. Singular variants are only observed in one patient.

Pathogenic variants in five genes alone accounted for almost half (48.8%) of our solved cases: *USH2A* (40/256, 15.6%), *ABCA4* (29/256, 11.3%), *EYS* (30/256, 11.3%), *PRPH2* (16/256, 6.25%), *CYP4V2* (10/256, 3.91%) (Fig. 3). The similarity between our findings and that of those reported in other studies involving Chinese cohorts [6, 7, 12, 29–31] can be explained by the predominance of Chinese patients in our cohort. We observed this similarity on the variant level as well. *EYS* variants were found only in Chinese patients, and the most common variant by far was c.6416 G > A (p.Cys2139Tyr) (20/30, 66.7%), similar to the prevalence in other Chinese cohorts [32], distantly followed by c.8107 G > T (p.Glu2703Ter) (8/30, 26.7%). The majority of the patients with a causative *CYP4V2* variant detected (8/10, 80%) had the founder variant c.802-8_810del17insGC [31] — in two patients this was biallelic, in four as compound heterozygous alleles with c.992 A > C (p.His331Pro), and in two patients as compound heterozygous with novel variants. The majority of the patients with *USH2A* variants were Chinese (36/40, 90.0%), with the most common variants being c.2802 T > G (p.Cys934Trp) (14/40, 35.0%) followed by c.8559-2 A > G and c.15178 T > C (p.Ser5060Pro) (both 4/40, 10.0%). Variants c.2802 T > G (p.Cys934Trp) and c.8559-2 A > G have been reported as the most common pathogenic variants in populations of Chinese ancestry [12, 32]. The demographics of *ABCA4* diagnosis were more diverse, where *ABCA4* is more commonly found in patients of Malay (6/17, 35.3%) and Indian (6/26, 23.1%) ancestries compared to its frequency among patients of Chinese ancestry (16/198, 8.1%), however, the sample size was not sufficient to observe variant-level trends. The founder variant found in UK population [33] *PRPH2* c.514 C > T (p.Arg172Trp) was also present in our Chinese patients as the most common *PRPH2* variant (4/15, 26.7%), followed by c.533 A > G (p.Gln178Arg) (3/15, 20.0%). Otherwise, pathogenic variants commonly observed in IRD cohorts of European descent were not present in our cohort nor have been described in other Asian cohorts [12, 34].

### Genotype-Phenotype concordance

**Fig. 4:**
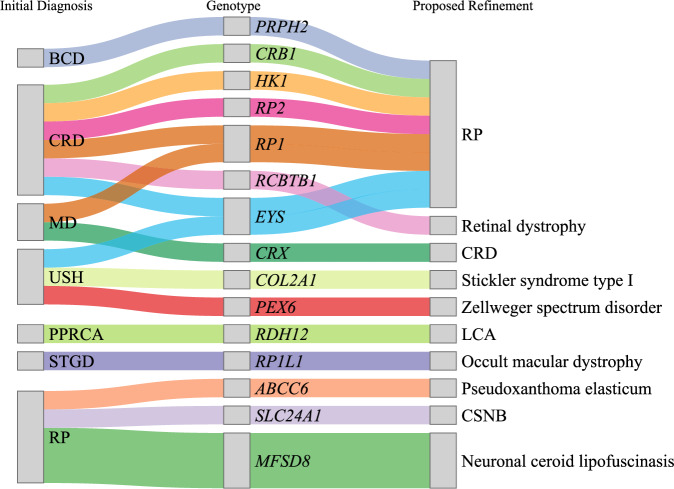
Possible refinement of initial clinical diagnosis based on genotyping information. From left to right: initial clinical diagnosis, molecular diagnosis, and proposed refined diagnosis.

We next examined the concordance between pathogenic variants identified through WES and the initial clinical diagnosis. For each IRD gene, we obtained a list of relevant clinical diagnoses through a combination of the Human Phenotype Ontology database (v2024-01-16) [35], RetNet [36], and PanelApp. Overall, 91.2% of the molecular diagnosis were in genes which corresponded with the clinical diagnosis, with certain phenotypes having 100% concordance, e.g. Retinoschisis (RS), Vitelliform dystrophy (VD), or Pattern dystrophy (PD) (Table 3). However, there were a few instances where molecular findings clarified the phenotypes, and this was not limited to a particular clinical phenotype (Fig. 4). The IRD conditions that benefited from molecular genotyping tended to be rare. For example, there were two patients who had been initially diagnosed with Usher syndrome based on the clinical presentations. However, molecular analysis for one patient identified a pathogenic variant in *PEX6*, which is associated with peroxisomal diseases, specifically Zellweger spectrum disorder [37], and another was found to carry a *COL4A1* variant associated with Stickler syndrome.

**Table 3:** The concordance of genotype with the initially diagnosed clinical phenotypes. Phenotypes carried by less than three probands are grouped under ‘Others’.

Clinical Diagnosis	Concordant (%)
Retinitis pigmentosa	120/125 (96.00%)
Cone/cone-rod dystrophy	22/29 (75.86%)
Stargardt-like disease	27/28 (96.43%)
Usher syndrome	11/14 (78.57%)
Bietti crystalline dystrophy	9/10 (90.00%)
Macular dystrophy	6/8 (75.00%)
Leber congenital amaurosis	6/6 (100.00%)
Bardet-Biedl syndrome	5/5 (100.00%)
Vitelliform dystrophy	5/5 (100.00%)
Retinoschisis	4/4 (100.00%)
Choroideremia	3/3 (100.00%)
Pattern dystrophy	3/3 (100.00%)
Others	8/9 (88.89%)

### Kinship analysis

When describing diagnostic yield and relative frequencies of pathogenic variants, there is a risk of closely-related individuals present in a cohort inflating observations. This is particularly relevant with IRD conditions where families may have multiple affected individuals and may seek care at different times. While family history is captured at study intake, this may not fully identify familial links among study participants. To reduce this bias, we used genetic kinship analysis on a part of the cohort in order to identify related individuals, with the purpose of creating an analysis cohort of unrelated individuals. We were also interested to determine the degree to which relatedness is missed when only self-reported familial associations are used. We discovered nine pairs of participants (18/515, 3.5%) with first- or second-degree kinship who were recruited yet not recorded as a family. Furthermore, of those, seven had no recorded family history information. Half of the newly discovered relationships were removed at random, reducing the total number of participants to 506. As expected, pairs of patients sharing the same variant are more related than patients who do not (median kinship coefficient −0.000735 vs −0.0239, *p* < 2.2e-16, Wilcoxon test). The inclusion of expected and unexpected relatives could result in overestimating the frequency of novel variants, as well as inaccurately inflating diagnostic yield and family history-related statistics.

## Discussion

Our study provides insights into the genetic architecture of IRDs in a sizable and diverse Asian population, with representation from East Asian, South Asian, and Southeast Asian ancestries, reinforcing several important determinants of diagnostic yield that can inform clinical practice. First, we provide clear evidence that genetic testing should be prioritized for patients with an earlier age of symptom onset or diagnosis. Consistent with prior studies across various populations [29, 30, 38–40], we observed a striking inverse correlation between diagnostic yield and age of clinical presentation, with patients diagnosed below age 20 having a 5.12-fold increase in odds ratio to receive molecular diagnosis compared to patients diagnosed above age 60. A possible reason is that age-related degradation and environmental causes of retinal damage that accumulate with age tend to obfuscate the diagnosis of IRDs in patients presenting at an older age.

Our finding that the presence of family history markedly improves the chances of obtaining a molecular diagnosis [4, 39, 41, 42] is an intuitive one, yet it is nevertheless important to quantify this effect to provide accurate pretest counseling and manage expectations appropriately. An accurate family history is often difficult to obtain and interpret, and in many societies, due to cultural and political circumstances, much of the population may not have the extended family to draw data on [7, 43]. Furthermore, molecular diagnosis is not contingent on family history, a reasonably high percentage of people who received molecular diagnosis do not also have family history [44]. However, its presence should be considered whenever available, and in resource-limited settings, priority for genetic testing should be considered for patients with more affected relatives.

In most cases, a patient’s diagnosis of IRD had a singular causal gene; however, the genotype-phenotype relationships are not necessarily exclusive, with several genes implicated in more than one clinical phenotype. Our findings are consistent with several genes being associated with the same clinical presentation, demonstrating the substantial phenotypic overlap of IRD genes. In the rare situation where there exists discordance between clinical presentation and molecular diagnosis, these tended to be early stages of syndromic RP prior to the emergence of extra-ocular symptoms. In such cases, for example our patient with the PEX6 variant, the patient greatly benefits from a molecular diagnosis as this enables appropriate management (e.g., referral to organ-specific specialists for review or screening).

The prospect of treatment for IRDs is promising with the number of genetic and gene-independent therapies undergoing clinical trials [45] and the commercial release of a *RPE65*-based therapy voretigene neparvovec (Luxturna; Spark Therapeutics Inc., Philadelphia, PA, USA). As most IRD research has involved patients of European ancestry, gene therapy development has primarily targeted variants frequently seen in that population, which may not be representative for other populations. For instance, *RPE65* accounts for 5-10% LCA cases in individuals of European descent [45], yet in other populations it is estimated to contribute less, for example1.7-5% in Indian or Chinese cohorts [46–48]. EDIT-101 (Editas Medicine, Massachusetts, USA) is a CRISPR-Cas9 based therapy targeting *CEP290*:c.2991+1655 A > G variant and commonly found in LCA patients of European ancestry [49]; however, the presence of this variant has not been identified in Chinese, Japanese, or Korean cohort studies [50]. *USH2A* is commonly associated with IRD in both East Asian and European cohorts [6–8, 12, 22, 29, 30, 32], however the most prevalent variants European populations are not significantly found in East Asian populations, and vice versa [12, 32]. With the size of *USH2A* and *ABCA4* each exceeding the carrying capacity of the frequently used adeno-associated virus (AAV) for gene therapy development, base-editing or similar strategies that target only a small portion of the gene may be a more viable strategy. Variants that are common in Asians are yet to be targeted in gene therapy trials. Without directed attention at Asian-specific gene therapy research, there may be disparities in access to gene-based treatments for IRD.

This study has several limitations. The application of WES does not capture all the intronic, structural, or copy number variants. Furthermore, regions with high GC bias are usually poorly covered, for example RPGR ORF15, in which most pathogenic *RPGR* mutations lie. To account for this, a small subset of samples with suspected RP or CRD were sent for specialized ophthalmic genetic testing which includes *RPGR* ORF15, which in turn detected six of the seven total pathogenic *RPGR* variants seen in this study. Despite this, our observed frequency of *RPGR* mutations is likely to be an underestimation. For patients where parental DNA was unavailable, we were unable to confirm that pairs of P/LP and/or VUS variants in recessive genes were in trans. However, where phasing was possible, we were able to confirm that 90% of such pairs were indeed in trans. As our patient demographics are representative of the Singapore population, our cohort is highly skewed towards patients of Chinese ancestry, which means that gene or variant frequencies observed in patients of other ancestries may be less robust. In addition, our variant prioritization algorithms may have filtered out common hypomorphic variants that result in late-presenting IRDs, although a search for known variants, e.g. *ABCA4* p.Asn1868Ile yielded nothing. Finally, this cohort was recruited from a tertiary hospital for adult patients and therefore may not be representative of all syndromic cases, which generally present at an earlier age, or retinal dystrophy conditions with childhood onset.

In summary, our study provides insights into optimizing the diagnostic utility of genetic testing for IRDs, enhancing the accuracy of genetic counseling for patients, and highlights opportunities to develop more inclusive targeted therapies.

## Supplementary information

Supplementary Note 1

Supplementary Table 2

Supplementary Table 1

Supplementary Legend

## Data Availability

All data generated or analysed during this study are included in this published article and its supplementary information files.
