# Supplementary material for: Determinants of diagnostic yield in a multi-ethnic Asian inherited retinal disease cohort
Source: Eur J Hum Genet. 2025 Mar 21;33(12):1627–35. doi: 10.1038/s41431-025-01833-w (PMC12669701; doi:10.1038/s41431-025-01833-w)
Supplement: Supplementary file 1 — Supplementary Note 1 [file 41431_2025_1833_MOESM1_ESM.docx]

# Variant Classification

Classification of candidate variants was performed according to ACMG/AMP (American College of Medical Genetics/Association for Molecular Pathology) guidelines^1^. Additional details for provided for the following criteria:

1. PVS1: All potential loss-of-function (LOF) variants were evaluated according to guidelines set forth by the ClinGen Sequence Variant Interpretation (SVI) workgroup^2^. For variants predicted to escape nonsense-mediated decay (NMD), the strength was modified to PVS1_Strong if > 10% of the protein was truncated, or if at least two 1-star ClinVar PLP or one 2-star ClinVar PLP variant could be found 3’ of the variant.
2. PS2: For *de novo* evidence, we evaluated primary literature for *de novo* occurrences. Points were calculated according to ClinGen SVI guidelines^3^, and a strength of Supporting, Moderate, Strong or Very Strong assigned based on total points.
3. PS3: When evaluating functional evidence, we examined primary literature and evaluated the experimental assays to determine if 1) appropriate positive and negative controls, 2) appropriate cell lines and 3) sufficient replicates were used.
4. PS4: Applied according to ACMG/AMP guidelines^1,4^. If the variant is rare and there are insufficient cases to calculate statistically meaningful relative risk (RR) or odds ratio (OR), we assign a modified strength of Moderate or Strong to variants where multiple unrelated patients shared the same phenotype and the variant was absent in a control population.
5. PM2: We applied a strength of Supporting instead of Moderate as suggested by ClinGen^5^.
6. PM3: When evaluating *in trans* observations, we used ClinGen SVI guidelines^6^ for assigning points. Depending on total points, a strength of Supporting, Moderate, Strong or Very Strong was assigned.
7. PM5: We applied PM5 with Moderate strength if there was a different missense change in the same codon reported in ClinVar with a review status of at least two stars. If more than one such missense change exists, a strength of Strong was applied.
8. PP1: We examined primary literature to assess evidence for co-segregation using recommendations set for by Jarvik and Browning^7^, and modified strength from Supporting to Moderate or Strong depending on strength of co-segregation.
9. PP3: We used REVEL scores to evaluate *in silico* pathogenicity and modified the strength from Supporting to Moderate and Strong of the REVEL score exceeded 0.773 or 0.932 respectively, as calibrated by the ClinGen SVI workgroup^8^.
10. PP4: We did not use PP4, except for *RS1*, which is very specific for the phenotype of retinoschisis and *CHM*, which is specific for choroideremia.
11. PP5 - We did not directly use PP5. Instead, where ClinVar/LOVD entries exist, we evaluated supporting evidence and primary literature and assigned relevant ACMG criteria.

**Case Resolution**

Case resolution depended on whether the variant(s) were found in autosomal dominant (AD) or autosomal recessive (AR) or X-linked (XL) gene, in compound heterozygous (CompHet), homozygous (Hom), heterozygous (Het), or hemizygous (Hemi) alleles.

| **Inheritance** | **Zygosity** | **Variant Classification(s)** | **Genetic Analysis** |
| --- | --- | --- | --- |
| AD/XL | Hom/Het/Hemi | VUS-FP | Probably Solved |
| AD/XL | Hom/Het/Hemi | LP/P | Solved |
| AR | Hom | VUS-FP | Probably Solved |
| AR | Hom | LP/P | Solved |
| AR | CompHet | VUS/VUS-FP +LP/P | Probably Solved |
| AR | CompHet | VUS-FP + VUS-FP | Probably Solved |
| AR | CompHet | LP/P + LP/P | Solved |

# Bibliography

1. Richards, S. *et al.* Standards and guidelines for the interpretation of sequence variants: A joint consensus recommendation of the American College of Medical Genetics and Genomics and the Association for Molecular Pathology. *Genet. Med. Off. J. Am. Coll. Med. Genet.* **17**, 405–424 (2015).

2. Abou Tayoun, A. N. *et al.* Recommendations for interpreting the loss of function PVS1 ACMG/AMP variant criterion. *Hum. Mutat.* **39**, 1517–1524 (2018).

3. Biesecker, L. G. & Harrison, S. M. SVI Recommendation for De Novo Criteria (PS2 & PM6). https://clinicalgenome.org/site/assets/files/3461/svi_proposal_for_de_novo_criteria_v1_1.pdf (2021).

4. Harrison, S. M., Biesecker, L. G. & Rehm, H. L. Overview of Specifications to the ACMG/AMP Variant Interpretation Guidelines. *Curr. Protoc. Hum. Genet.* **103**, e93 (2019).

5. Biesecker, L. G. & Harrison, S. M. Recommendation for Absence/Rarity Criterion PM2. https://www.clinicalgenome.org/site/assets/files/5182/pm2_-_svi_recommendation_-_approved_sept2020.pdf.

6. Biesecker, L. G. & Harrison, S. M. Recommendation for in trans Criterion PM3. https://clinicalgenome.org/site/assets/files/3717/svi_proposal_for_pm3_criterion_-_version_1.pdf.

7. Jarvik, G. P. & Browning, B. L. Consideration of Cosegregation in the Pathogenicity Classification of Genomic Variants. *Am. J. Hum. Genet.* **98**, 1077–1081 (2016).

8. Pejaver, V. *et al.* Calibration of computational tools for missense variant pathogenicity classification and ClinGen recommendations for PP3/BP4 criteria. *Am. J. Hum. Genet.* **109**, 2163–2177 (2022).
