# Supplementary material for: Determinants of diagnostic yield in a multi-ethnic Asian inherited retinal disease cohort
Source: Eur J Hum Genet. 2025 Mar 21;33(12):1627–35. doi: 10.1038/s41431-025-01833-w (PMC12669701; doi:10.1038/s41431-025-01833-w)
Supplement: Supplementary file 4 — Supplementary Legend [file 41431_2025_1833_MOESM4_ESM.docx]

Supplementary Table 1. Table of proband data that were used for the analysis in this paper. This table includes the Pubmed IDs of studies that were used as functional studies evidence (PS3) or segregation calculation (PP1).

Supplementary Table 2. Table of PanelApp derived gene panel.

Supplementary Note 1. Details of variant classification process and decision table for considering cases as solved, probably solved, or not solved.
